# Reorganization of the Intact Somatosensory Cortex Immediately after Spinal Cord Injury

**DOI:** 10.1371/journal.pone.0069655

**Published:** 2013-07-29

**Authors:** Desire Humanes-Valera, Juan Aguilar, Guglielmo Foffani

**Affiliations:** Hospital Nacional de Parapléjicos, Servicio de Salud de Castilla-La Mancha, Toledo, Spain; Hertie Institute for Clinical Brain Research, University of Tuebingen, Germany

## Abstract

Sensory deafferentation produces extensive reorganization of the corresponding deafferented cortex. Little is known, however, about the role of the adjacent intact cortex in this reorganization. Here we show that a complete thoracic transection of the spinal cord immediately increases the responses of the intact forepaw cortex to forepaw stimuli (above the level of the lesion) in anesthetized rats. These increased forepaw responses were independent of the global changes in cortical state induced by the spinal cord transection described in our previous work (Aguilar et al., J Neurosci 2010), as the responses increased both when the cortex was in a silent state (down-state) or in an active state (up-state). The increased responses in the intact forepaw cortex correlated with increased responses in the deafferented hindpaw cortex, suggesting that they could represent different points of view of the same immediate state-independent functional reorganization of the primary somatosensory cortex after spinal cord injury. Collectively, the results of the present study and of our previous study suggest that both state-dependent and state-independent mechanisms can jointly contribute to cortical reorganization immediately after spinal cord injury.

## Introduction

When a major lesion of the nervous system – such as stroke, amputation or spinal cord injury - interrupts the normal flow of sensory inputs from the body to the brain, the corresponding sensory function is obviously dramatically affected. In addition to the direct loss of function, sensory deafferentation produces extensive long-term reorganization of brain structures up to the cortex [1–5]. Long-term cortical reorganization might contribute to recovery of spared functions [6–10], but excessive or aberrant reorganization can produce real sensations that don’t correspond with the external objective reality, such as phantom sensations [11,12] and neuropathic pain [13–17]. To understand the early mechanisms underlying cortical reorganization after deafferentation is therefore critical in order to develop timely interventions to properly manage its pathological consequences and optimize recovery [18].

Cortical reorganization after deafferentation is typically described in terms of the deafferented cortex becoming more responsive to stimulation of the surrounding intact body regions. In a recent study [19], we indirectly pointed toward a different – and much overlooked (but see e.g. 20) – cortical reorganization, characterized by the intact cortex adjacent to the deafferented cortex becoming more responsive to stimulation of intact body regions. Specifically, after complete thoracic transection of the spinal cord in anesthetized rats, the intact forepaw cortex immediately became more responsive to stimuli delivered to the forepaw, above the lesion level. Importantly, these increased forepaw responses correlated with a slower and overall more silent cortical spontaneous activity, suggesting that they could simply reflect the change in the cortical state induced by the deafferentation, due to the known state dependence of cortical somatosensory responses [21–26]. Whether immediately after spinal cord injury the intact cortex undergoes any reorganization that does not depend on global changes in cortical state remains unknown.

To disentangle possible contributions of state-dependent vs state-independent mechanisms, we performed electrophysiological recordings in both the forepaw and hindpaw cortex in anesthetized rats, recording cortical responses to forepaw stimuli under three conditions: (1) intact rats at the same level of anesthesia as in our previous study [19]; (2) after delivering additional anesthesia to mimic the cortical state change produced by spinal cord injury, still keeping the spinal cord intact; (3) within 1-h after performing a thoracic transection of the spinal cord in animals that were already deeply anesthetized. The strategy is to compare the responses to stimuli delivered while the cortex is in a similar state both before and after spinal cord transection, in order to uncover any state-independent reorganization of the intact forepaw cortex.

## Materials and Methods

**Figure 1:**
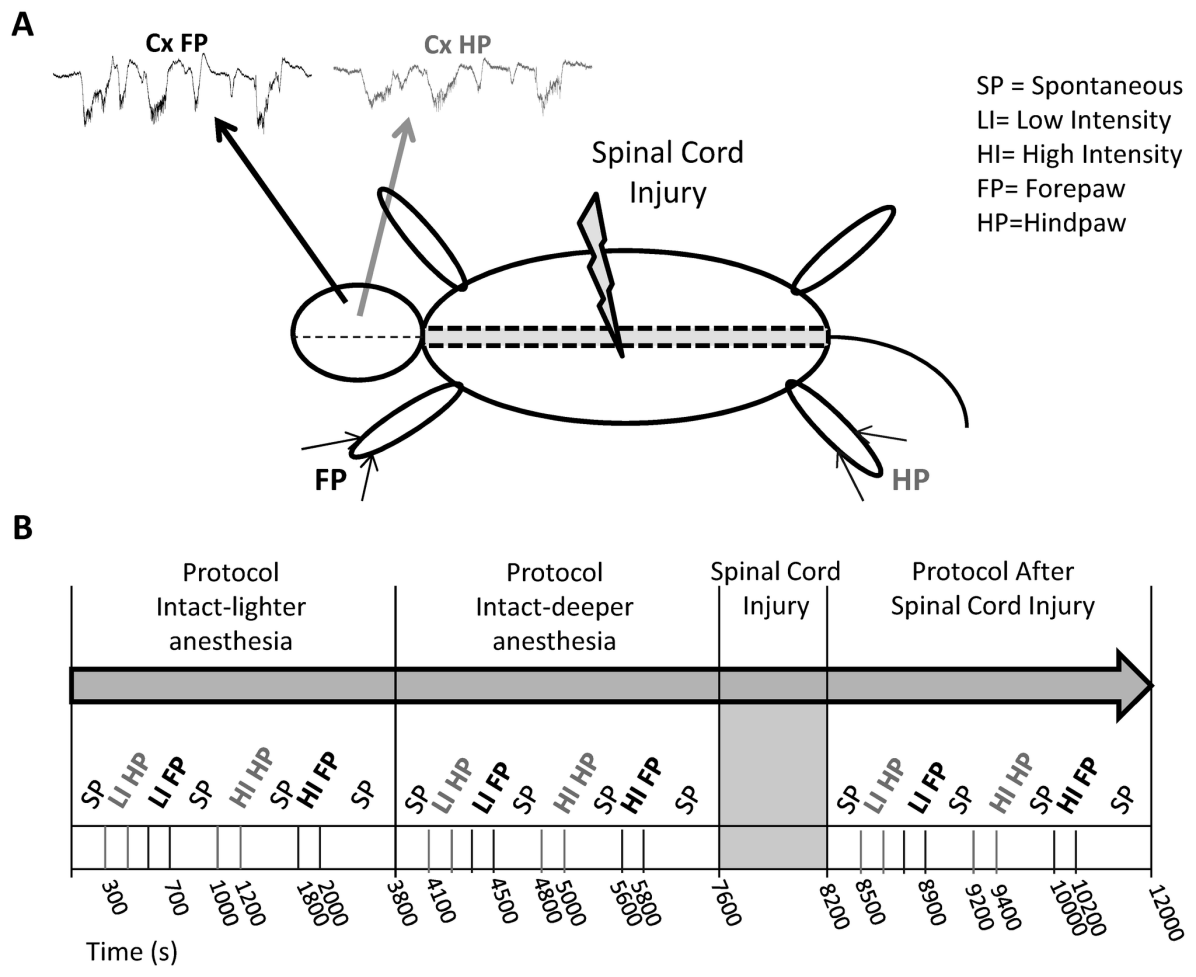
Experimental protocol. (A) Extracellular recordings were made in the forepaw (FP) and hindpaw (HP) representations of the primary somatosensory cortex (Cx) in urethane-anaesthetized rats. Complete transection of the spinal cord was performed at thoracic level (T9-T10). (**B**) We studied both the spontaneous activity and the responses evoked by electrical stimuli delivered to the hindpaw and forepaw at low intensity (0.5 mA) and high intensity (5 mA). The first pre-lesion protocol was performed with intact spinal cord and lighter anesthesia. We then administered an additional dose of urethane to obtain a consistent state of slow-wave activity. In this state we performed the second pre-lesion protocol, with intact spinal cord and deeper anesthesia. We finally performed the complete transection of the spinal cord and after 30 min we started the post-lesion protocol with the spinal cord transected.

**Figure 2:**
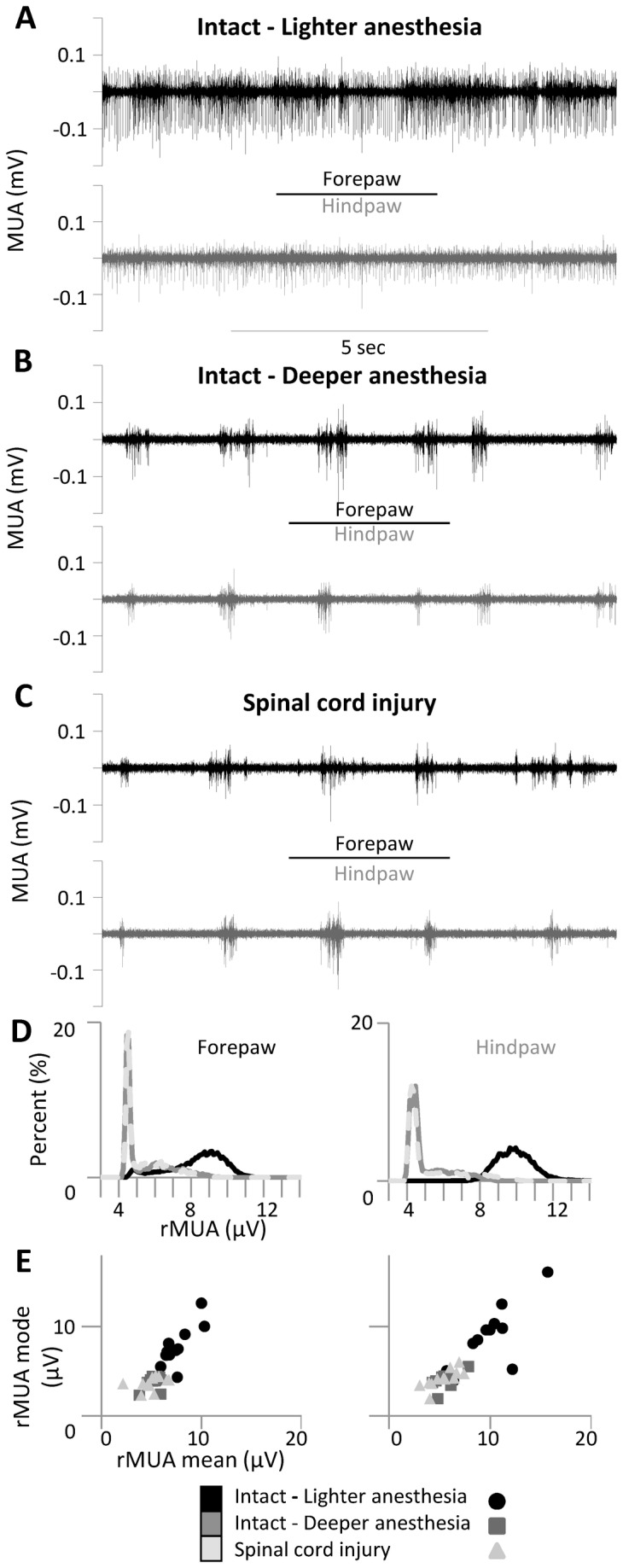
Spontaneous activity. (**A**–**C**) Spontaneous multi-unit activity (MUA) simultaneously recorded in the forepaw cortex (black) and hindpaw cortex (gray) under three different conditions in a representative animal: (**A**) intact animal under lighter anesthesia, (**B**) intact animal under deeper anesthesia and (**C**) immediately after (<1 hour) complete thoracic transection of the spinal cord. (**D**) Distributions of rectified MUA (rMUA) in a representative animal and (**E**) scatter plots of rMUA mean and mode in all animals. Cortical spontaneous activity decreased after additional anesthesia but was not further affected by spinal cord transection.

Experiments were performed following the rules of the International Council for Laboratory Animal Science, European Union regulation 2010/63/EU and approved by the Ethical Committee for Animal Research of the Hospital Nacional de Parapléjicos (Toledo, Spain). A total of 20 male Wistar rats were used, divided in 2 experimental groups: 1) in the main experiment in 12 animals we performed extracellular recordings from the intact forepaw cortex and the deafferented hindpaw cortex before and immediately after complete thoracic transection of the spinal cord (Figure 1); 2) in a control experiment in 8 animals we performed the recordings before and immediately after “sham” lesion. The general experimental approach (anesthesia, surgery, extracellular recordings and peripheral stimulation) is similar to our previous studies [19,27,28].

### Experimental protocol

Animals were anesthetized with urethane (1.5g/kg i.p.). The body temperature was kept constant (36.5 ºC) using an automatically controlled heating pad. Animals were placed in a stereotaxic frame (SR-6 Narishige Scientific Instruments, Tokio, Japan). A laminectomy was performed at thoracic level (T9-T10) keeping the dura mater intact and covered with saline to protect the area. The skull was exposed and a craniotomy was performed on the right side of the midline over the primary somatosensory cortex (antero-posterior: 1 to -4; medio-lateral: 1-5 [29]) and the cisterna magna was opened to decrease brain pressure and thus improve the stability of the recordings. Small incisions in the dura mater were performed to allow the recordings electrodes to be lowered into the cerebral cortex. Once the electrodes were placed in the hindpaw and forepaw representations of the primary somatosensory cortex, we performed the first pre-lesion protocol, recording evoked responses and spontaneous activity with intact spinal cord and lighter anesthesia (stage III-3 [30,31]). Additional doses of urethane (<1/6 of the induction dose) were then administered as necessary in order to obtain a consistent state of deep slow-wave activity. In this state we performed the second pre-lesion protocol, with intact spinal cord and deeper anesthesia (stage III-4 [30,31]). We finally performed the complete transection of the spinal cord with a scalpel blade. Immediately after transection, few pulses of electrical stimulation of the hindpaw at very-high intensity (10mA) were applied in order to confirm that no physiological responses were evoked in the cortex by stimuli delivered below the level of the lesion. The complete cut of the spinal cord was visually confirmed under the surgical microscope by the total separation of the borders. About 30 min after the transection, we started the post-lesion protocol, recording evoked responses and spontaneous activity with the spinal cord transected.

### Electrophysiology

Recordings were obtained using tungsten electrodes with 4-5 MΩ impedance at 1000 Hz (TM31C40KT and TM31A50KT of WPI, Inc, Sarasota, FL, USA). Two electrodes were stereotaxically lowered in the infragranular somatosensory cortex, one in the forepaw area (antero-posterior: 0.5mm; medio-lateral: 4mm; depth 1.1-1.6mm) and the other one in the hindpaw area (antero-posterior: -1mm; medio-lateral: 2.5mm; depth: 1.1-1.6mm) following the coordinates of [32]. In 10 of 12 experiments, a third electrode was also lowered in the forepaw area between the two original electrodes (anterior-posterior: 0mm). We specifically targeted the infragranular layers because they are (1) the cortical layers expressing maximal convergence of excitatory and inhibitory inputs, both local and long-range (2), the main origin of cortical outputs, and (3) the layers where the majority of active states originate [33–36]. The infragranular cortex is thus particularly appropriate to investigate at the network level cortical changes after deafferentation. Once the electrodes were fixed in place, they were not moved throughout the entire experiment. All recordings were pre-amplified in DC mode, low-pass filtered (< 3kHz) and amplified using a modular system (Neurolog; Digitimer Ltd.). Analog signals were converted into digital data at 20 kHz sampling rate and 16-bit quantization using a CED power 1401 (Cambridge Electronics Design, Cambridge, UK) controlled by Spike2 software (v6, Cambridge Electronics Desing, Cambridge, UK). Signals were stored in a hard disk of a PC for posterior analysis.

### Peripheral stimulation

Electrical pulses were applied using bipolar needle electrodes located subcutaneously in the wrist of the forepaw and of the hindpaw, one pole in each side of the paw. The rationale for this stimulation was to activate all types of somatosensory fibers originating within the paws, including tactile, proprioceptive and nociceptive fibers. The protocol consisted of a total of 100 pulse stimuli with duration of 1ms and frequency of 0.5Hz. Two different intensities were applied: low-intensity (0.5mA) and high-intensity (5mA). Low-intensity stimuli were intended to activate only a fraction of the available fibers, mainly low-threshold primary fibers running through the lemniscal pathway, from the dorsal columns to the brainstem [27,37]. High-intensity stimuli were intended to activate the maximum number of fibers, including high-threshold primary fibers that make synapse in the dorsal horns of the spinal cord, in turn activating the spinothalamic tract [27,37].

### Control experiments

We also performed a set of “sham” experiments (n=8) in which the spinal cord remained intact after the laminectomy for the entire duration of the experiment. Besides the absence of spinal cord lesion, the experimental protocol was the same as in the transected animals, but sham animals were studied only under deep anesthesia.

### Data analysis

#### Spontaneous activity

Spontaneous activity was studied in recordings at least 150-s long, performed immediately after the low-intensity stimulation and immediately before the high-intensity stimulation. To quantitatively evaluate the level of cortical spontaneous activity, we extracted the rectified multi-unit activity (rMUA) by band-pass filtering the raw signals at high frequencies (300-3000 Hz) and rectifying the resulting signal. The rMUA is a good measure of cortical state, as it correlates with the membrane potential of adjacent intracellularly recorded neurons [24]. Under lighter anesthesia the somatosensory cortex typically was in a relatively active state, characterized by a high probability of the rMUA to be at high voltage. Under deeper anesthesia, after delivering additional urethane still keeping the spinal cord intact, the somatosensory cortex switched to a global state of slow-wave oscillations (<1 Hz [38,39]), characterized by a typical bimodal distribution of the rMUA, representing down-states in a narrow peak at low voltages and up-states in a broader peak at higher voltages, similarly to the membrane potential of intracellularly recorded neurons [24]. Immediately after transection of the spinal cord, with the animals already under deep anesthesia, the somatosensory cortex remained in a global state of slow-wave oscillations (<1 Hz) at a similar level of spontaneous activity, as evidenced by an overlapping rMUA distribution. The overall level of cortical spontaneous activity was therefore assessed by the mean and the mode of the rMUA (the mode of the rMUA is the most likely value, i.e. the highest peak of the rMUA distribution). We also measured the peak frequency of the rMUA spectrum, which we have previously shown to be very sensitive to subtle changes in the frequency of up/down oscillations within the same state of slow-wave activity [28].

#### Evoked responses

Local field potential (LFP) responses were obtained by averaging across stimuli the raw signals recorded from the electrodes. The amplitude of LFP responses was evaluated as the absolute value of the negative peak in the average response. Single-trial analyses were performed by quantifying the average rectified MUA in the 50ms before each stimulus and the peak-to-peak amplitude of the LFP response to the subsequent stimulus. Furthermore, stimuli were visually classified as delivered during DOWN or UP cortical states based on both LFP and MUA signals on a trial-by-trial basis according to the following criteria: (1) if both LFP and MUA signals showed no activity for at least 100 ms before the stimulus, the stimulus was classified as delivered during a DOWN state; (2) if both LFP and MUA showed activity immediately before the stimulus, the stimulus was classified as delivered during an UP state (3) if neither of the above criteria was verified, the stimulus remained unclassified.

Multi-unit responses were obtained by band-pass filtering the raw LFP signals at high frequencies (300-3000Hz), rectifying the resulting signal, and averaging across stimuli. The magnitude of rMUA responses was evaluated as the area of the response in the first 50 ms post-stimulus, after subtracting the background. The duration of post-response inhibition was measured in the rMUA response as the time interval from the end of the first excitatory response to the onset of the subsequent activation, using only stimuli occurring during silent states.

### Statistical analyses

Changes in cortical spontaneous activity were evaluated separately entering the mean and the mode of the rMUA into two-way repeated-measures ANOVAs, with TIME as first factor with three levels (lighter anesthesia, deeper anesthesia, and after spinal transection) and ELECTRODE as second factor with two levels (forepaw cortex and hindpaw cortex). The same two-way ANOVA was used for the peak of the rMUA spectrum, but because this measure is often ambiguous during lighter anesthesia, only two levels in the TIME factor were considered (deeper anesthesia vs after spinal cord transection). Note that one animal was recorded only under deeper anesthesia and after spinal cord transection, so it was treated as missing value under lighter anesthesia.

Changes in LFP responses in the forepaw cortex to forepaw stimuli were evaluated with two-way repeated-measures ANOVAs, with TIME as first factor with three levels (lighter anesthesia, deeper anesthesia, and after spinal cord transection) and STIMULUS as second factor with two levels (low-intensity and high-intensity). Changes in the duration of post-response inhibition to high-intensity forepaw stimuli were evaluated with a one-way repeated-measures ANOVA on the TIME factor. Changes in cortical LFP responses separating stimuli as delivered during UP or DOWN cortical states were evaluated with a two-way repeated-measures ANOVA, with TIME as first factor with two levels (before and after spinal transection) and STATE as second factor with two levels (UP and DOWN), using only high-intensity stimuli.

In the experiments with the third electrode, changes in LFP responses in the forepaw cortex to high-intensity forepaw stimuli were also evaluated with two-way repeated-measures ANOVA, with TIME as first factor with two levels (deeper anesthesia vs after spinal cord transection) and ELECTRODE as second factor with two levels (forepaw cortex 1 and forepaw cortex 2).

In the control experiments with sham animals, we performed the same two-way ANOVAs as is the main experiments, but with only two levels in the TIME factor (before sham lesion, after sham lesion).

Changes in rMUA responses in the hindpaw cortex to high-intensity forepaw stimuli were evaluated with a one-way repeated-measures ANOVA on the TIME factor.

Tukey Honest Significant Difference test was used for all post-hoc comparisons. Additional comparisons between means, percentages and correlations were performed with t-tests, two-proportion test and Pearson’s correlation coefficient, as indicated in the text. All results were considered significant at p<0.05.

## Results

**Figure 3:**
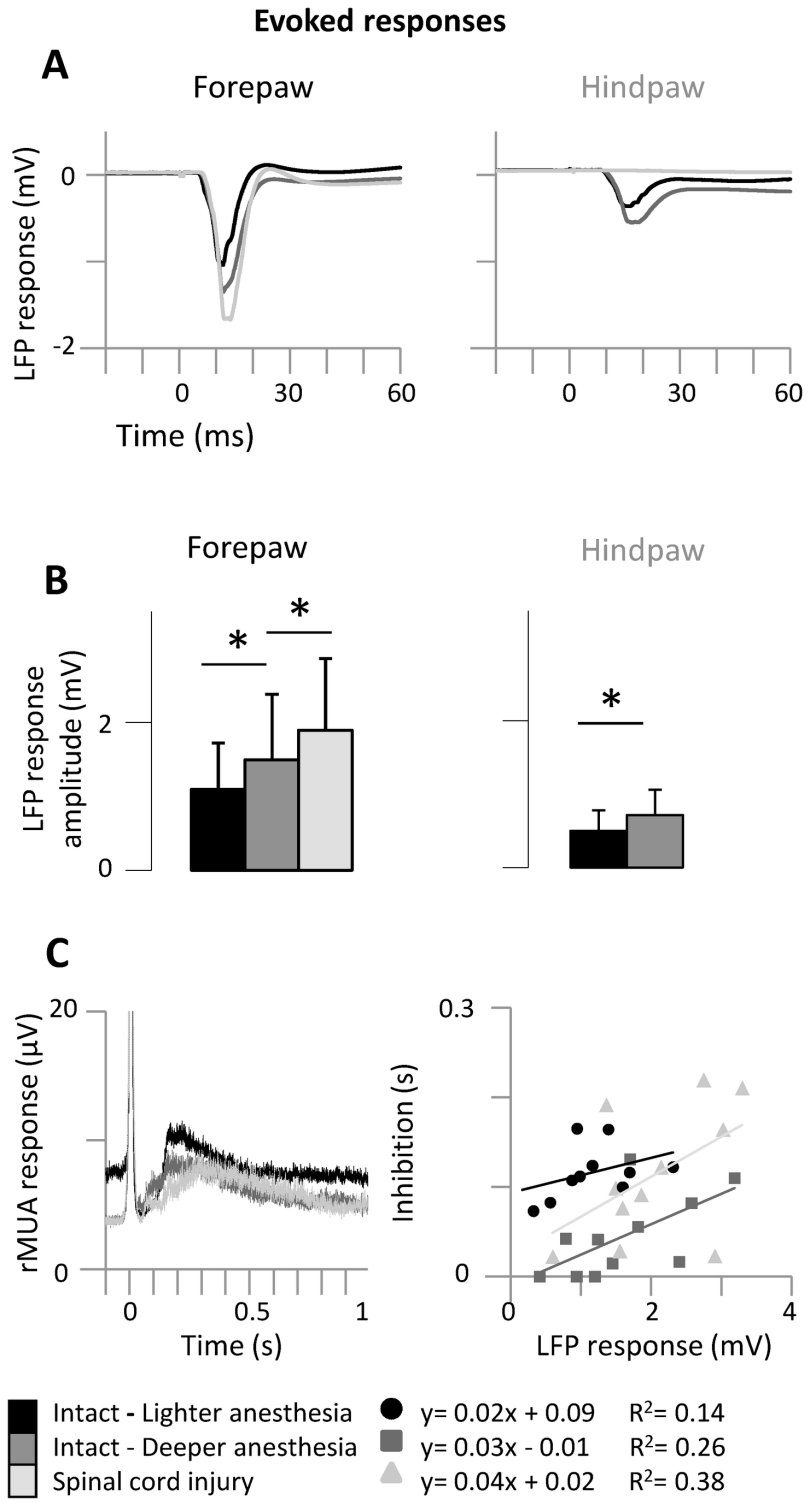
Average responses evoked in the intact forepaw cortex by forepaw stimuli. (**A**) Grand average of local field potential (LFP) responses evoked by high-intensity (5mA) stimuli delivered either to the contralateral forepaw (left) or hindpaw (right) (n=12 animals). (**B**) Corresponding measures of response amplitude. Bars represent means, error bars are standard deviations. Asterisks indicate significant differences between adjacent conditions (see text). (**C**) Post-response inhibition (left). Grand-averages of rMUA responses evoked in forepaw cortex by forepaw stimuli. Note that rMUA responses are truncated at 20µV in order to focus on post-response inhibition (right). Correlation between amplitude of LFP responses (x-axis) and duration of post-response inhibition (y-axis) in all animals. Even without a global change in cortical spontaneous activity, the responses evoked in the intact forepaw cortex by forepaw stimuli and the associated post-response inhibition both increased immediately after thoracic transection of the spinal cord.

We simultaneously recorded local field potentials (LFPs) and multiunit activity (MUA) from the hindpaw representation and the forepaw representation of the primary somatosensory cortex (n=12 rats) in response to somatosensory stimuli delivered to the contralateral forepaw and hindpaw, under three experimental conditions in the same animals: (1) intact animals under relatively light anesthesia (stage III-3 [30,31]; Figures 2A), (2) intact animals under deeper anesthesia (stage III-4 [30,31]; Figures 2B), (3) immediately after spinal cord transection (Figure 2C). Statistical analyses – on the mean and the mode of the rMUA distribution (Figure 2D) – confirmed the change in cortical state between lighter and deeper anesthesia (two-way ANOVA, time factor: F(2,20)>30.6, p<0.0001; Tukey: p<0.0002) and the absence of additional changes in global cortical state after spinal cord transection under deep anesthesia (Tukey p>0.88) (Figure 2E). To confirm the absence of global changes in cortical state after spinal cord transection under deep anesthesia, we also measured the peak frequency of rMUA spectrum, which did not change after spinal cord transection in both the intact forepaw cortex (pre: 0.57±0.13 Hz; post: 0.57±0.14 Hz) and the deafferented hindpaw cortex (pre: 0.58±0.14 Hz; post: 0.50±0.12 Hz) (time factor: F(1,10)=0.8, p=0.40). We will first describe the changes in the evoked responses, and we will subsequently jointly analyze changes in spontaneous activity and in the evoked responses on a single-trial basis.

### Average responses evoked in the intact forepaw cortex by forepaw stimuli

Under lighter anesthesia, the LFP responses evoked in the forepaw cortex by forepaw stimuli displayed greater amplitude than the LFP responses evoked in the hindpaw cortex by hindpaw stimuli (Figure 3A,B black; Table 1), which is consistent with our previous works [19,40,41]. The cortical state change induced by deeper anesthesia (Figure 3A,B dark gray) reproduced the main neurophysiological effects of the cortical state change induced by spinal cord injury in our previous study [19,42]: the amplitude of the LFP responses evoked in the forepaw cortex by high-intensity forepaw stimuli markedly increased (two-way ANOVA, interaction factor: F(2,20)=14.1, p=0.0001; Tukey: p=0.0020; Table 1). Immediately after transection of the spinal cord, LFP responses evoked in the hindpaw cortex by hindpaw stimuli were abolished, as expected (Figure 3A right). Conversely, LFP responses evoked in the forepaw cortex by forepaw stimuli again markedly increased (Figure 3A left, light gray; Table 1), even without any cortical state change involved. As above, these increased amplitudes were specifically observed in response to high-intensity stimuli (Tukey: p=0.0064; Figure 3B left), but they did not reach significance in response to low-intensity stimuli (Table 1). Similar results were obtained with the rMUA responses to high-intensity forepaw stimuli (one-way ANOVA: F(2,20)=12.9, p=0.0003), which tended to increase under deeper anesthesia (Tukey: p=0.0798) and significantly increased after spinal cord transection (p=0.0306).

**Table 1:** Amplitudes of LFP cortical responses before and immediately after complete thoracic transection of the spinal cord or sham lesion.

	LIGHTER ANESTHESIA INTACT		DEEPER ANESTHESIA INTACT		AFTER SPINAL CORD TRANSECTION	
	Spinal cord transection (n=12)					
	HP	FP	HP	FP	HP	FP
Low-intensity stimuli (mV)	0.24±0.21	0.24±0.08	0.29±0.21	0.28±0.14		0.38±0.26
High-intensity stimuli (mV)	0.50±0.28	1.09±0.63	0.71±0.35	1.49±0.89		1.89±0.97
				DOWN: 1.67±0.98		DOWN: 2.12±1.04
				UP: 0.91±0.56		UP: 1.43±0.83
	Sham (n=8)					
			HP	FP	HP	FP
Low-intensity stimuli (mV)			0.15±0.12	0.23±0.15	0.13±0.10	0.22±0.21
High-intensity stimuli (mV)			0.43±0.26	1.34±0.95	0.51±0.34	1.32±0.73

Values are means ± standard deviations.

Interestingly, the state-*dependent* increase of LFP response amplitude after additional anesthesia was associated with a *decrease* in the duration of the post-response inhibition (from 116.4±30.0ms to 44.8±45.7ms; one-way ANOVA: F(2,18)=8.3, p=0.0028; Tukey: p=0.0063), whereas the state-*independent* increase of LFP response amplitude after spinal cord transection was mirrored by an *increase* in the duration of the post-response inhibition (from 44.8±45.7ms to 113.4±74.2ms; Tukey p=0.0069), as evident in the rMUA responses (Figure 3C left). In fact, when the cortex was in slow-wave activity the LFP response and the duration of post-response inhibition were positively correlated (Pearson: R>0.5, n=11; Figure 3C right). It is important to note that after spinal cord injury the regression line between LFP response (x-axis) and post-response inhibition (y-axis) was clearly above the same regression line before spinal cord injury (deeper anesthesia), suggesting that the increased post-response inhibition after spinal cord injury is not simply a consequence of the increased response, but instead reflects altered intra-cortical inhibition after the lesion.

### Relation between cortical spontaneous activity and evoked responses on a single-trial basis

**Figure 4:**
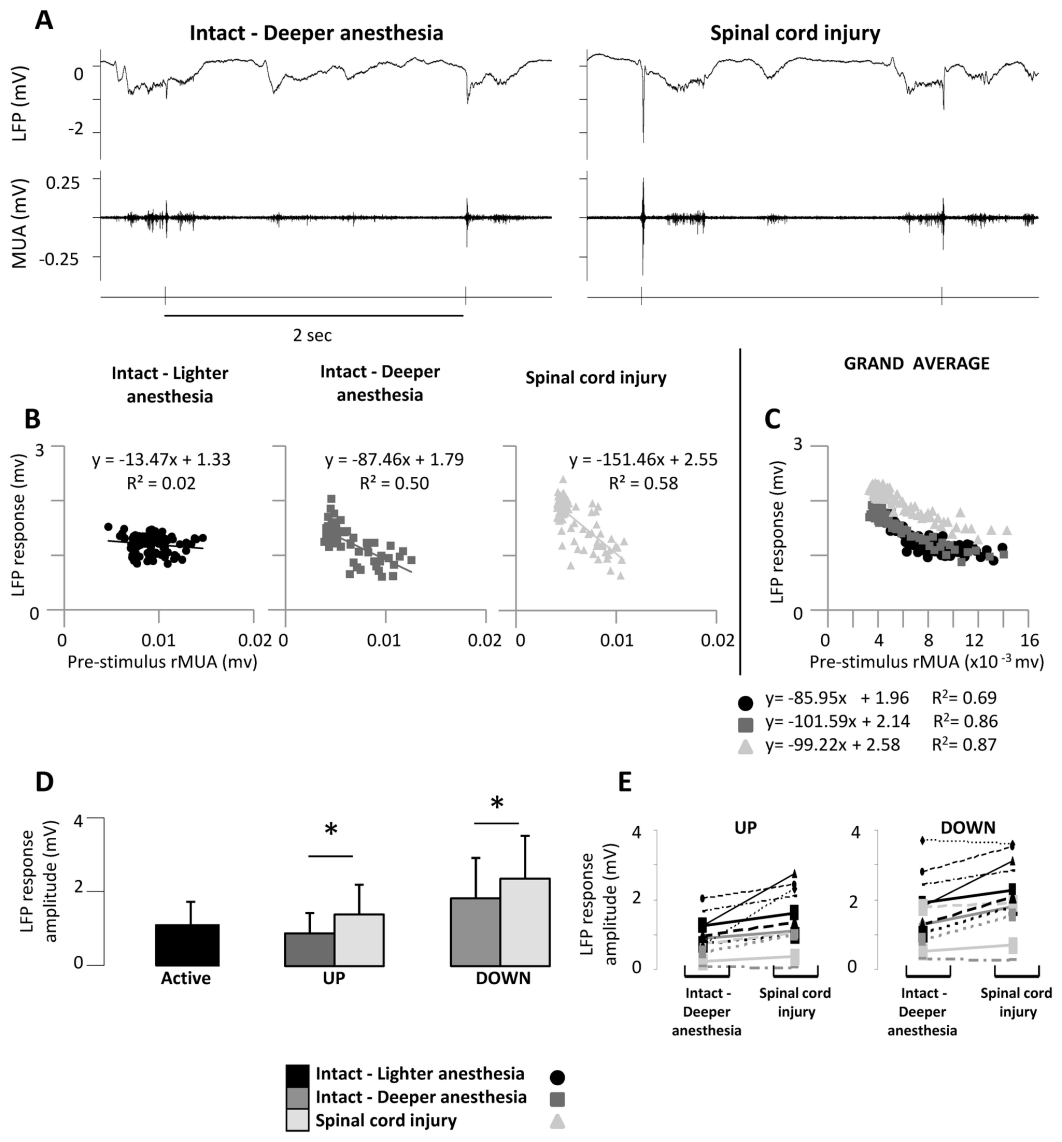
Relation between cortical spontaneous activity and evoked responses on a single-trial basis. (**A**) Responses evoked in the forepaw cortex by high-intensity forepaw stimuli in a representative animal under deep anesthesia (left) and immediately after complete thoracic transection of the spinal cord (right). The plots show LFP recordings (upper traces) and MUA recordings (lower traces) around two single-trial stimuli delivered during UP and DOWN states. (**B**,**C**) Joint single-trial analysis of spontaneous activity (mean rMUA in the 50 ms pre-stimulus, x-axes) and responses evoked in the forepaw cortex by high-intensity forepaw stimuli (LFP amplitude, y-axes). Each value corresponds to an individual stimulus, delivered every 2 s. (**B**) Representative animal and (**C**) corresponding grand-average of all animals. (**D**,**E**) Amplitudes of evoked responses after separating the stimuli based on whether they occurred during down states (low values of pre-stimulus rMUA) or up states (high values of pre-stimulus rMUA). (**D**) Pooled measures from all animals, showing the amplitudes of the responses in the different cortical states. Bars represent means, error bars are standard deviations. (**E**) Corresponding variability between animals. When we separately analyzed the rMUA pre-stimulus and the single-trial LFP responses, we essentially confirmed and extended the results reported in Figure 2 and Figure 3: (i) change in cortical state induced by anesthesia, (ii) no additional change in cortical state induced by spinal cord injury under deep anesthesia, (iii) state-dependent increase in the responses evoked in the forepaw cortex by forepaw stimuli after anesthesia, (iv) state-independent increase in the responses evoked in the forepaw cortex by forepaw stimuli after spinal cord transection. The responses evoked in the intact forepaw cortex by forepaw stimuli increased immediately after thoracic transection of the spinal cord both when stimuli where delivered during UP or DOWN cortical states.

In order to gain additional insights into the network mechanisms of the increased responses evoked in the forepaw cortex by high-intensity forepaw stimuli, we reanalyzed the data on a single-trial basis. Both before and after spinal cord transection, when a stimulus occurred while the cortex was in an active state, it evoked a smaller response compared to a stimulus that occurred while the cortex was in a silent state (Figure 4A), consistently with our previous observations [19]. To take into account this source of variability within stimulation protocols, we focused the attention on the relation between the spontaneous activity immediately before each stimulus – measured by the mean rMUA in the 50ms pre-stimulus – and the corresponding single-trial LFP response. When we jointly analyzed the pre-stimulus rMUA and the single-trial LFP response, we could separate state-dependent vs state-independent changes in cortical evoked responses (Figure 4B,C). In intact animals the amplitude of the LFP response was inversely correlated to the pre-stimulus rMUA: with lighter anesthesia most responses clustered at higher pre-stimulus rMUA with lower LFP response amplitude (Figure 4C, black; Pearson R=-0.83, n=100); with deeper anesthesia many responses clustered at lower pre-stimulus rMUA with higher LFP response amplitude (Figure 4C, dark gray; R=-0.93, n=100). After spinal cord transection, LFP responses increased at all values of pre-stimulus rMUA, confirming a state-independent change (Figure 4C, light gray; R=-0.93, n=100).

Because during cortical slow-wave oscillations the lower values of pre-stimulus rMUA correspond to the down states and the higher values of pre-stimulus rMUA correspond to the up states, we repeated the statistical analyses previously performed on the average responses (see Figure 3) after separating the stimuli into two sets: (1) stimuli delivered during silent (DOWN) cortical states and (2) stimuli delivered during active (UP) cortical states (Figure 4D,E). The increased forepaw responses after spinal cord transection were observed both with high-intensity stimuli delivered during DOWN states and with high-intensity stimuli delivered during UP states (two-way ANOVA, time factor: F(1,11)=22.5, p=0.0006; interaction factor: F(1,11)=0.06, p=0.81; Table 1). Because most of the state-dependent variability of cortical somatosensory responses can be predicted by the spontaneous activity immediately preceding the stimuli, these single-trial analyses allowed us to separate state-independent vs state-dependent cortical reorganization.

### Additional experiments: spatial consistency and controls

**Figure 5:**
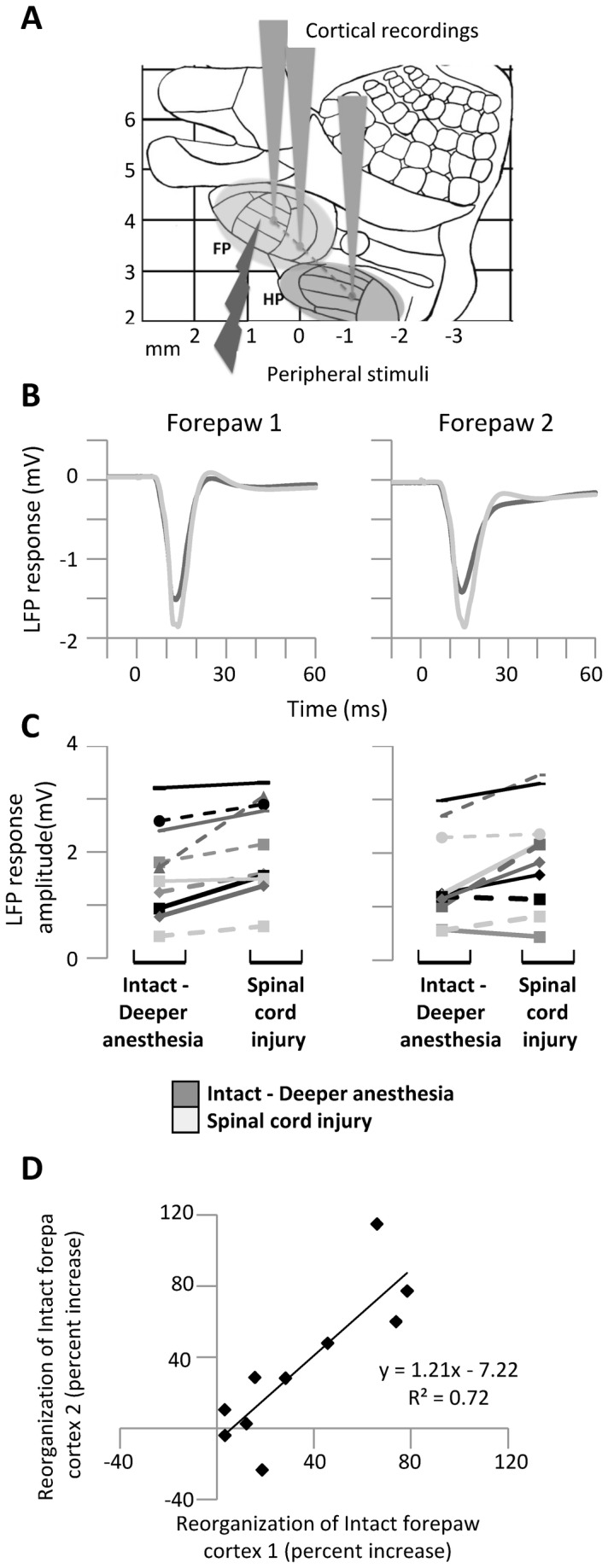
Spatial consistency within the forepaw cortex. (**A**) Diagram representing the experimental protocol: on a schematic map of the rat primary somatosensory cortex, the gray cones represent the recording locations in the forepaw area (FP) and hindpaw area (HP) and the light arrow represents the peripheral electrical stimuli delivered to the forepaw. (**B**) Grand average LFP responses evoked in the two electrodes within the forepaw cortex by high-intensity forepaw stimuli before and immediately after spinal cord transection under deep anesthesia. (**C**) Corresponding variability of LFP response amplitude between animals. (**D**) Correlation in the reorganization of the intact forepaw cortex as measured by percent increase of the responses evoked by high-intensity forepaw stimuli (100*after transection/before transection-1) at the two forepaw electrodes. Both electrodes in the forepaw cortex show a similar state-independent increase in the responses evoked by high-intensity forepaw stimuli after spinal cord transection, confirming the spatial consistency of our results.

In order to verify that the increased responses to forepaw stimuli after spinal transection were not contingent on the precise location of the electrode in the forepaw cortex, in 10 of 12 experiments a third electrode was lowered in the forepaw cortex between the two original electrodes (Figure 5A). Both electrodes showed a similar increase in the responses evoked in the forepaw cortex by high-intensity forepaw stimuli after spinal cord transection (two-way ANOVA, time factor: F(1,9)=13.6, p=0.0050; interaction factor: F(1,9)=0.02, p=0.89; Figure 5B,C). In fact the percent increase of the responses (100*after transection/before transection-1) was correlated between the two electrodes (Pearson R= 0.85, n=10; Figure 5D), confirming the spatial consistency of our results within the forepaw cortex.

Finally, to exclude the possibility that our results were not due to the spinal lesion (e.g. to exclude possible plasticity effects due to cortical slow-wave activity, see 43), we verified that the increase of forepaw responses did not occur in a series of control experiments (n=8) in rats that were recorded under deep anesthesia before and after ‘sham’ lesion (two-way ANOVA, time factor: F(1,7)<0.1, p=0.90; interaction factor: F(1,7)<0.01, p=0.96; Table 1).

Overall, the above results show that immediately after spinal cord transection the responses of the intact forepaw cortex to forepaw stimuli (above the level of the lesion) increase even in the absence of global changes in cortical state, reflecting a state-independent immediate cortical reorganization – in addition to the state-dependent reorganization we previously described – after spinal cord injury.

### Responses evoked in the deafferented hindpaw cortex by forepaw stimuli

Once established that state-independent functional reorganization occurs in the intact forepaw cortex immediately after spinal cord transection, we investigated whether state-independent reorganization also occurred in the deafferented hindpaw cortex. To this end, we used the rectified multi-unit activity (rMUA) – which reflects the overall spiking activity around the electrode – to quantify the responses evoked in the deafferented hindpaw cortex by forepaw stimuli in the same animals for which the forepaw responses were analyzed above (n=12).

**Figure 6:**
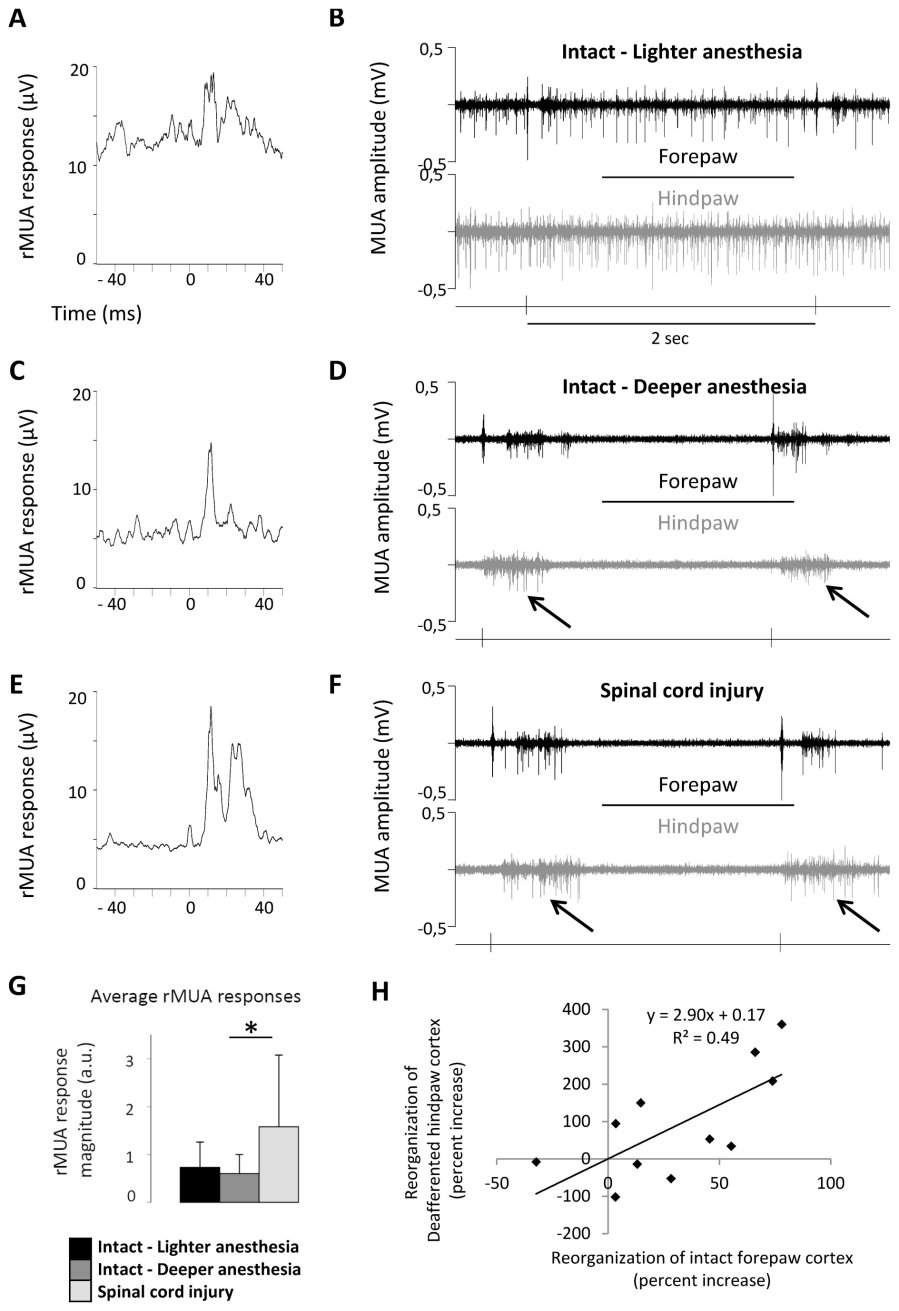
Responses evoked in the deafferented hindpaw cortex by forepaw stimuli. (**A**–**F**) Responses evoked in the hindpaw cortex by stimuli delivered to the contralateral forepaw under three different conditions in a representative animal: (**A**,**B**) intact animal under lighter anesthesia, (**C**,**D**) intact animal under deeper anesthesia and (**E**,**F**) immediately after (<1 hour) complete thoracic transection of the spinal cord. Left plots (**A**,**C**,**E**) show the short-latency responses, as measured by the rectified MUA (rMUA), evoked in the hindpaw cortex by high-intensity (5mA) stimuli delivered to the contralateral forepaw. Right plots (**B**,**D**,**F**) show MUA recordings in the forepaw cortex (black) and in the hindpaw cortex (gray) for two single-trial stimuli in the different conditions. Arrows in **D** and **F** indicate the UP states triggered by forepaw stimuli in the hindpaw cortex, generating long-latency activations. (**G**) Pooled measure of rMUA response magnitude of short-latency responses from all animals (n=12). Short-latency responses evoked in the deafferented hindpaw cortex by forepaw stimuli increased immediately after thoracic transection of the spinal cord due to state-independent mechanisms, whereas long-latency activations were purely state-dependent. (**H**) Reorganization in the deafferented hindpaw cortex (as measured by the percent increase of short-latency rMUA responses evoked in the hindpaw cortex by forepaw stimuli) and reorganization in the intact forepaw cortex (as measured by the percent increase of LFP responses evoked in the forepaw cortex by forepaw stimuli) were correlated.

As expected, we observed two types of “responses”: (1) short-latency responses (<50 ms) and (2) long-latency activations (>50 ms). Short-latency responses are typical somatosensory responses – of the same type as the “non-homologous responses” we described in [40] – observed in most animals [19]. Indeed, the magnitude (background-subtracted) of short-latency responses, evoked by high-intensity forepaw stimuli in the deafferented hindpaw cortex, did not change between lighter and deeper anesthesia (one-way ANOVA: F(2,20)=4.7, p=0.0208; Tukey: p=0.90) but significantly increased after spinal cord transection (Tukey: p=0.0254; Figure 6A–G).

Long-latency activations are instead due to active states triggered by the stimuli [19]. As expected, long latency activations were not observed when the cortex was active under lighter anesthesia and were consistently observed in the cortical state of slow-wave oscillations, both under deeper anesthesia and after spinal cord injury (Figure 6B,D,F). Considering only high-intensity stimuli occurring during silent cortical states, long-latency activations – measured as the probability of active states triggered by the stimuli to be observed the hindpaw cortex – did not differ before (0.83±0.14) and after (0.80±0.24) spinal cord transection (paired t-test: p=0.49).

These results suggest that long-latency activations depend exclusively on the global state of the somatosesory cortex, whereas the increased short-latency responses represent state-independent functional reorganization of the deafferented hindpaw cortex immediately after spinal cord transection. Interestingly, reorganization of the deafferented hindpaw cortex (as measured by the percent increase of short-latency rMUA responses evoked in the hindpaw cortex by forepaw stimuli) and reorganization of the intact forepaw cortex (as measured by the percent increase of LFP responses evoked in the forepaw cortex by forepaw stimuli) were positively correlated across animals (Pearson: R=0.70, n=11; one animal was excluded from this analysis as outlier), suggesting that they represent two complementary views of the same immediate cortical reorganization after spinal cord injury (Figure 6H).

## Discussion

Our main result is that a complete thoracic transection of the spinal cord immediately increases the responses of the intact forepaw cortex to forepaw stimuli (above the level of the lesion) in anesthetized rats. These increased forepaw responses are independent of the global changes in cortical state induced by the spinal cord transection described in our previous work [19], as they were seen both when the cortex was in a silent state (down-state) or in an active state (up-state). The increased responses in the intact forepaw cortex correlate with increased responses in the deafferented hindpaw cortex, suggesting that they could represent different points of view of the same state-independent functional reorganization of the primary somatosensory cortex immediately after spinal cord injury.

### State-dependent vs state-independent cortical reorganization

We previously showed that thoracic spinal cord transection immediately changes the state of the brain – slowing it down – and that this state change is at least partly responsible for the increased responses evoked in the intact forepaw cortex by forepaw stimuli [19]. In our model, spinal cord injury reduces anesthetic requirements [42]. This observation is consistent with the sedative effects induced by spinal anesthesia in animals and patients, most likely due to the loss of somatosensory inputs to the arousal centers in the brainstem (discussed in 42). Even though the underlying mechanisms are likely different, the cortical state changes induced by spinal cord injury in our model are similar to the cortical state changes induced by anesthesia. We were therefore able to reproduce the state-dependent increase of forepaw responses by simply delivering additional anesthesia.

The experimental strategy of bringing animals into a state of deep anesthesia before the spinal cord injury was critical to disentangle the contribution of state-independent mechanisms to the increased responses in the intact forepaw cortex immediately after the spinal injury. Even though we cannot exclude more subtle changes in the local state of the somatosensory cortex, several complementary observations support the existence of state-independent reorganization (i.e. reorganization that does not rely on a change in the global state of the somatosensory cortex): (1) with the cortex already in slow-wave activity, the overall level of cortical spontaneous activity did not change after spinal transection (as measured by the rMUA mean, mode and spectral peak frequency; Figure 2); (2) state-dependent increases of forepaw responses after additional anesthesia were associated with decreased post-response inhibition, whereas state-independent increases of forepaw responses after spinal transection were associated with increased post-response inhibition (Figure 3); (3) performing single-trial analyses, the increased forepaw responses were observed both when the cortex was in down-states and in up-states (Figure 4). Down states, in particular, represent a very controlled cortical state, with virtually no spontaneous firing at all, and are thus particularly appropriate to isolate state-independent cortical reorganization in the intact forepaw cortex.

Forepaw stimuli evoke two main types of responses in the hindpaw cortex [19]: (1) short-latency responses (<50 ms), which correspond to classical somatosensory responses, and (2) long-latency activations (>50 ms), which correspond to up-states triggered by the stimuli. Here we show that immediately after thoracic transection of the spinal cord the magnitude of short-latency responses increases exclusively by state-independent mechanisms, whereas the increased probability to observe long-latency activations, reported in our previous study [19], is exclusively state-dependent (Figure 6). Whether state-dependent long-latency activations contribute to the long-term cortical reorganization observed after spinal cord injury in fMRI studies [10,44,45] will deserve further investigation. In any case, our findings suggest that the immediate reorganization of the primary somatosensory cortex classically observed after peripheral injuries [46] – and more recently after stroke [47,48] – can be generalized to spinal cord injury.

### Possible mechanisms of state-independent cortical reorganization

Several non-exclusive mechanisms can contribute to cortical reorganization after deafferentation, including growth of new connections due to axonal sprouting [49], unmasking of latent connections due to reduction of intra-cortical inhibition [50,51], or changes in neuronal intrinsic properties. Despite recent evidence of axonal sprouting in the deafferented cortex as early as few hours after retinal lesions in monkeys [52] and after whisker plucking in rats [53], the time frame of our reorganization seems too short for axonal sprouting to substantially contribute to our results. However, reduction of *tonic* intra-cortical inhibition is ruled out here, because the responses in the intact forepaw cortex increased even if the stimuli were delivered during silent cortical states, when there is no tonic inhibition to be reduced [38,54,55]. Our data show that even the evoked intra-cortical post-response inhibition is actually increased after deafferentation. Whereas the state-dependent decrease of post-response inhibition is consistent with decreased cholinergic neuromodulation, the state-independent increase of post-response inhibition seems consistent with *increased* noradrenergic neuromodulation [56,57]. This possible intriguing imbalance between cholinergic and noradrenergic neuromodulation after spinal cord injury deserves further investigation. Overall, our state-independent cortical reorganization could in principle be explained by a change in the excitation/inhibiton ratio. Indeed, reduction of dendritic spines and axonal boutons of inhibitory interneurons in the deafferented cortex – and to a less degree in the adjacent cortex – was recently observed as early as 6 h after retinal lesions in mice [58]. Heterogeneous spine loss has also been reported as early as 3 days after spinal cord injury in mice [59]. It is therefore tempting to propose that cortical structural changes represent the consequence – rather than the cause – of functional reorganization.

One might wonder about the possible mechanistic role of urethane anesthesia in our results. On the one hand urethane anesthesia is considered a good model of natural sleep [60]. Slow-wave activity during sleep increases sensory evoked responses in subsequent wake periods [43], but not during slow-wave activity itself [43], as confirmed by our control experiments with sham lesions. On the other hand, urethane anesthesia can affect the intrinsic properties of cortical pyramidal neurons in vitro but has little or no effects at synaptic level [61], suggesting that altered LTP/LTD mechanisms – typical of other anesthetics [62–64] – are unlikely to play a major role in our experimental model.

Finally, we cannot exclude that at least part of the reorganization we observed at cortical level could reflect reorganization occurring at subcortical level. The fact that our results were significant only with high intensity stimuli – which likely activate both the dorsal columns and the spinothalamic tract [27,37] – and not with low-intensity stimuli – which likely activate only the dorsal columns [27,37] – suggests that the subcortical interaction between lemniscal and paralemniscal systems [65,66] might play a role in our experimental model [45]. Nonetheless, subcortical reorganization could in principle occur either in the thalamus [45,67–69], in the brainstem [67,69–71], or even within the spinal cord [72,73].

Independently of the exact cortical/subcortical mechanisms, the increased cortical responsiveness reported here represents the overall state-independent reorganization of the somatosensory system immediately after thoracic spinal cord transection.

### Pathophysiological significance

The great majority of studies investigating reorganization in the somatosensory system after peripheral or spinal cord injuries implicitly assume that intact structures do not undergo any reorganization. Our results clarify that this is not the case. Several previous findings conceptually support the possible long-term pathophysiological relevance of the immediate reorganization of the intact forepaw cortex reported here: (1) careful a posteriori examination of the figures/results of previous works on long-term cortical reorganization after thoracic spinal cord injury in rats [10,45] suggests that long-term reorganization of the intact forepaw cortex was likely present in the data; (2) above-normal activation of the intact motor cortex is observed in paraplegic patients moving their unaffected upper limb [20]; (3) similar above-normal activation of the intact face somatosensory cortex is observed in spinal cord injury patients following median nerve stimulation [74]; (4) both rats and patients can develop long-term hypersensitivity and pain well above the level of spinal cord injury [75]; (5) exercise-induced plasticity of the intact forelimb cortex is associated with greater reorganization of the deafferented hindlimb cortex in neonatally spinalized rats [8,76]. The intriguing correlation we observed between reorganization of the intact forepaw cortex and deafferented hindpaw cortex immediately after thoracic spinal cord transection could therefore be important not only to fully understand the early mechanisms that lead to long-term cortical reorganization, but also to develop timely interventions to properly manage the possible pathological consequences of such reorganization and to optimize recovery after spinal cord injury [18].

In conclusion, the results of the present study and of our previous study [19] collectively suggest that both state-dependent and state-independent mechanisms can jointly contribute to cortical reorganization immediately after thoracic spinal cord transection. This immediate functional reorganization could contribute to the cascade of mechanisms that lead to long-term cortical reorganization after spinal cord injury [77].
